# The risk of sexual dysfunction in Chinese women with recurrent pregnancy loss and the associated factors: a multicenter cross-sectional study

**DOI:** 10.1093/sexmed/qfae031

**Published:** 2024-05-26

**Authors:** Chuanjiao Liu, Yiduo Zhang, Qian Xu, Chen Yang, Yuanting Tang, Qiong Zhang, Xiumei Liu, Yangang Yue, Fan Yu

**Affiliations:** Department of Laboratory Medicine, Chengdu Qingbaijiang Maternal and Child Health Care Hospital, Qingbaijiang District, Chengdu 610300, Sichuan Province, China; Department of Laboratory Medicine, Chengdu Qingbaijiang Women’s and Children’s Hospital, West China Second University Hospital, Sichuan University, Chengdu 610300, Sichuan Province, China; Department of Laboratory Medicine, West China Second University Hospital, Sichuan University, Chengdu 610041, Sichuan Province, China; Key Laboratory of Birth Defects and Related Diseases of Women and Children, Sichuan University, Ministry of Education, Chengdu 610041, Sichuan Province, China; Department of Laboratory Medicine, Chengdu Qingbaijiang Maternal and Child Health Care Hospital, Qingbaijiang District, Chengdu 610300, Sichuan Province, China; Department of Laboratory Medicine, Chengdu Qingbaijiang Women’s and Children’s Hospital, West China Second University Hospital, Sichuan University, Chengdu 610300, Sichuan Province, China; Department of Laboratory Medicine, Chengdu Qingbaijiang Maternal and Child Health Care Hospital, Qingbaijiang District, Chengdu 610300, Sichuan Province, China; Department of Laboratory Medicine, Chengdu Qingbaijiang Women’s and Children’s Hospital, West China Second University Hospital, Sichuan University, Chengdu 610300, Sichuan Province, China; Department of Laboratory Medicine, West China Second University Hospital, Sichuan University, Chengdu 610041, Sichuan Province, China; Key Laboratory of Birth Defects and Related Diseases of Women and Children, Sichuan University, Ministry of Education, Chengdu 610041, Sichuan Province, China; Department of Obstetrics and Gynecology, Ziyang Maternal and Child Health Care Hospital, Ziyang 641300, China; Department of Obstetrics and Gynecology, Ziyang Maternal and Child Health Care Hospital, Ziyang 641300, China; Department of Laboratory Medicine, Chengdu Qingbaijiang Maternal and Child Health Care Hospital, Qingbaijiang District, Chengdu 610300, Sichuan Province, China; Department of Laboratory Medicine, Chengdu Qingbaijiang Women’s and Children’s Hospital, West China Second University Hospital, Sichuan University, Chengdu 610300, Sichuan Province, China; Department of Laboratory Medicine, West China Second University Hospital, Sichuan University, Chengdu 610041, Sichuan Province, China; Key Laboratory of Birth Defects and Related Diseases of Women and Children, Sichuan University, Ministry of Education, Chengdu 610041, Sichuan Province, China

**Keywords:** recurrent pregnancy loss, sexual dysfunction, risk factors, cross-sectional study

## Abstract

**Background:**

Recurrent pregnancy loss (RPL) is a severe traumatic event for women of childbearing age. However, the association between RPL and female sexual dysfunction was unknown.

**Aim:**

The study sought to investigate the association between RPL and sexual dysfunction, and to explore the risk factors of sexual dysfunction for RPL patients.

**Methods:**

A multicenter cross-sectional study involving both RPL patients and healthy women was performed in 3 different hospitals in West China from May 2021 to January 2023. Baseline information including sociodemographic data and disease histories were collected. The Female Sexual Function Index (FSFI) was used to assess the sexual function of participants.

**Outcomes:**

The main outcome was the proportion of women at increased risk of sexual dysfunction (total FSFI scores <26.55), and the secondary outcome was risk factors of sexual dysfunction in RPL patients.

**Results:**

A total of 233 RPL patients and 185 healthy women were included in this study. RPL patients had significantly lower total FSFI scores (median 31.7 [interquartile range, 26.6-33.5] vs 33.0 [interquartile range, 31.2-34.1]; *P <* .001) and a significantly higher risk of sexual dysfunction than healthy women (24.9% vs 8.6%; *P <* .001). Body mass index >24 kg/m^2^ (adjusted odds ratio [OR], 4.132; 95% confidence interval [CI], 1.902-8.976, *P <* .001), working >8 h/d (adjusted OR, 2.111; 95% CI, 1.020-4.369, *P =* .044), and unexplained RPL (adjusted OR, 3.785; 95% CI, 1.967-7.280, *P <* .001) were independent risk factors of sexual dysfunction for RPL patients.

**Clinical Implications:**

RPL patients, especially those patients with the previously mentioned risk factors, should be focused on the risk of sexual dysfunction, and appropriate preventions could be applied.

**Strength and Limitations:**

We explored the association between RPL and sexual dysfunction and explored the risk factors of sexual dysfunction among RPL patients for the first time, and the multicenter data increased the generalizability of results. However, the cross-sectional design did not provide an exact causal relationship between RPL and sexual dysfunction, and potential risk factors related to mental health were not investigated.

**Conclusion:**

RPL patients were at an increased risk of sexual dysfunction. Overweight, fatigue caused by work, and unexplained RPL were risk factors of sexual dysfunction for RPL patients.

## Introduction

Recurrent pregnancy loss (RPL) is defined as 2 or more times of consecutive spontaneous abortions before 24 gestational weeks, affecting about 1% to 2% of couples around the world.^1^^,^^2^ RPL is a highly heterogeneous condition with various maternal, paternal and fetal risk factors, including abnormal chromosomes in parents or fetus, endocrinological disorders, immune disorders, uterine abnormalities, thrombophilia, and male factors.^3^^,^^4^ Current diagnostic strategies can identify etiologic factors in only 50% of these patients, and other patients were defined as unexplained RPL.^5^ Unexplained RPL is a distressing problem for both patients and clinicians because there is no effective management strategy for these patients.^6^ It cannot be ignored that RPL has a variety of adverse impacts on the health of the affected patients. RPL patients had a higher risk of pregnancy complications, such as pre-eclampsia, stillbirth, and premature delivery, in the next pregnancy compared with healthy women.^7^^,^^8^ RPL was also associated with long-term health problems beyond pregnancy; for example, studies found that RPL was associated with an increased risk of cardiovascular disease and venous thromboembolism.^2^^,^^9^

Sexual dysfunction is a problem that can happen during any phase of the sexual response cycle, which includes desire, arousal, plateau, orgasm, and resolution.^10^ Sexual dysfunction prevents patients from experiencing satisfaction from sexual activity and causes serious adverse effects on the quality of life.^11^ Any number of medical conditions, including cancer, multiple sclerosis, heart disease, and rheumatologic diseases, as well as certain medications, including chemotherapy drugs, blood pressure medications, and antihistamines, can lead to female sexual dysfunction.^12^^,^^13^ However, the effects of RPL on female sexual dysfunction have not been investigated until now. Does RPL increase the risk of female sexual dysfunction? What are the risk factors for sexual dysfunction among RPL patients?

## Methods

### Participants recruitment

A multicenter, cross-sectional study was performed in 3 different hospitals in West China. RPL patients and healthy women were recruited into this study from May 2021 to January 2023 by 3 clinicians (C.L., Y.Z., and Q.Z.). RPL patients were recruited at the department of obstetrics in 3 hospitals, and the diagnosis of RPL was rechecked by a fourth author (X.L.) according to the European Society of Human Reproduction and Embryology guideline.^1^^,^^14^ Control women were recruited from the health examination center of Chengdu Qingbaijiang Maternal and Child Health Care Hospital, and the general health status and pregnancy histories were checked by 1 author (F.Y.). Only women without any history of pregnancy complications or adverse pregnancy outcomes were included in this study. In addition, to exclude the effects of marital status and pregnancy on results, only participants who were married and not pregnant during the investigation were finally included. Participants were divided into 2 groups, RPL and healthy women, according to the diagnosis of RPL or not. The Female Sexual Function Index (FSFI) was used to assess the risk of female sexual dysfunction. The manuscript was prepared according to the STROBE (Strenghtening the Reporting of Observational Studies in Epidemiology) statement.

### Participants selection

The participants were selected independently by 2 authors (C.L. and Y.Y.) according to the pre-established inclusion criteria. The inconsistency of participant selection between the 2 authors was discussed with a third author (F.Y.) until they reached an agreement finally.

The inclusion criteria for RPL patients were (1) age between 20 and 45 years; (2) RPL occurred after natural pregnancy, not after assisted reproductive technology; (3) without other diseases at present, including diagnosed physical and mental disorders; and (4) agreement to cooperate with the questionary investigation. The inclusion criteria for healthy women were (1) age between 20 and 45 years, (2) without other diagnosed physical and mental disorders at present, and (3) agreement to cooperate with the questionary investigation.

The exclusion criteria for all participants were (1) without sexual life in the recent 4 weeks and (2) missing important information, including sociodemographic information and clinical data that would be analyzed in this study.

### Sample size calculation

We calculated that we would need to enroll at least 130 participants in each group. Few research data related to the proportion of sexual dysfunction or FSFI scores in RPL patients or healthy women have been previously published. We assumed that the sample size was equal in the 2 groups, and the assumed proportions of participants at high risk of sexual dysfunction in the RPL group and healthy women were 25% and 10%, respectively. The level of statistical significance was set at 5% (α = 0.05) using a 2-sided test and 99% power (1 – β). We calculated the expected sample size by PASS software 2020 (NCSS, LLC).

### Baseline information collection

After participants were included in this study, their baseline information was collected immediately by 3 authors (Q.X., Y.Z., and X.L.). Age and body mass index (BMI) were collected via hospital visit records. Sociodemographic information of participants, including working hours per day, education background, smoking, alcohol consumption, residence, and household income (yuan/month) was collected by asking via WeChat or telephone. Disease histories of RPL patients were collected via hospital visit records, including causes of RPL, the times of spontaneous miscarriage, stillbirth, and induced abortion.

### Sexual function assessment

We used the FSFI to assess the sexual function of included participants. When participants were confirmed to be included in this study, they were invited to take part in a face-to-face interview. We explained the study’s content, how to fill out this tool, and any problems that they were concerned with in detail. If they felt uncomfortable with the content, they could withdraw from the study at any time. The FSFI is a powerful screening tool for assessing female sexual function, extensively used worldwide.^15^^,^^16^ The 19 items of the FSFI use a 5-point scale ranging from 1 to 5 with higher scores indicating greater levels of sexual functioning on the respective item. The sum of each domain score was first multiplied by a domain factor ratio (0.6 for desire, 0.3 for arousal, 0.3 for lubrication, 0.4 for orgasm, 0.4 for satisfaction, and 0.4 for pain), and subsequently got a total FSFI score. A total FSFI score of <26.55 was regarded as an increased risk of sexual dysfunction according to the acknowledged cutoff value.^17^

### Ethical approval

This study was approved by the Medical Ethics Committee of Chengdu Qingbaijiang Maternal and Child Health Care Hospital (No. 2021011), and participants provided written informed consent.

### Statistical analysis

Continuous variables with abnormal distributions were presented as median (interquartile range) and were compared by the Mann-Whitney test, and continuous variables with normal distributions were presented as mean ± SD and were compared by Student’s *t* test. Categorical variables were presented as frequency and percentage and were compared by chi-square test. If the frequency of equal or more than 1 cell was <5, Fisher’s exact test was used. Logistic regression analysis was performed to explore the independent effects of variables on the increased risk of sexual dysfunction. The outcomes were shown as odds ratio (OR) and 95% confidential interval (CI). A *P* value <.05 was regarded as statistically different. The statistical analyses were performed by SPSS software (version 23.0; IBM).

## Results

### Participant inclusion

**Figure 1:**
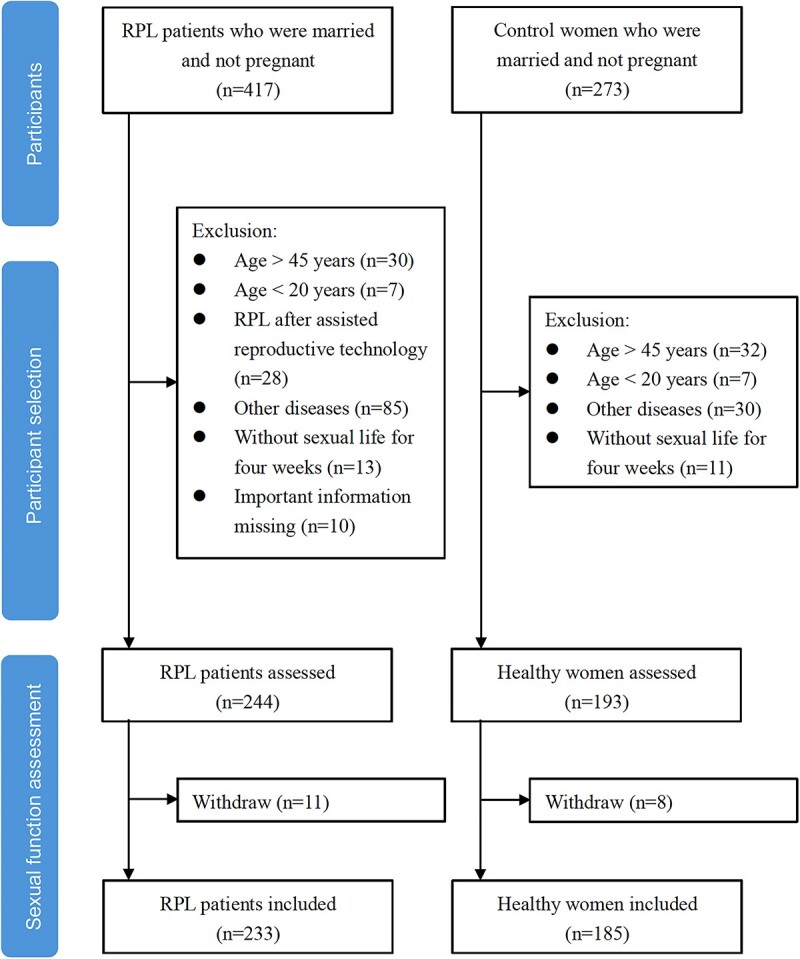
Flow chart of participant recruitment, selection, and sexual function assessment.

A total of 417 RPL patients and 273 control women who were married and not pregnant were recruited in this study. After participant selection, 244 RPL patients and 193 healthy women received sexual function assessment, and 11 RPL patients and 8 healthy women withdrew from the study subsequently. Finally, 233 RPL patients and 185 healthy women were included in this cross-sectional study. The flow chart of participant recruitment, selection, and sexual function assessment is shown in Figure 1.

### Baseline information

There were significant differences in age, educational background, and residence between included RPL patients and healthy women. The proportion ≥36 years of age was higher in RPL patients than in healthy women (21.9% vs 8.6%; *P <* .001). The frequency of lower education level (40.8% vs 23.2%; *P <* .001) and rural residence (10.3% vs 3.8%; *P =* .012) in RPL patients was significantly higher than those in healthy women. However, we did not observe a statistical difference in BMI, working hours per day, smoking, alcohol consumption, and household income between the 2 groups.

Of 233 included RPL patients, 59.2% were caused by parental or fetal factors, and 40.8% were diagnosed as unexplained RPL. Up to 37.2% of RPL patients experienced more than 2 spontaneous abortions, 14.2% of RPL patients had a history of stillbirth, and 66.1% of RPL patients had a history of induced abortion. These results are shown in Table 1.

**Table 1:** Baseline information of participants.

	RPL patients (n = 233)	Healthy women (n = 185)	*P* value
Age			
≤35 y	182 (78.1)	169 (91.4)	<.001
≥36 y	51 (21.9)	16 (8.6)	
BMI			
<18.5 kg/m^2^	23 (9.9)	17 (9.2)	.596
18.5-24 kg/m^2^	170 (73.0)	129 (69.7)	
>24 kg/m^2^	40 (17.1)	39 (21.1)	
Working hours			
≤8 h/d	181 (77.7)	151 (81.6)	.322
>8 h/d	52 (22.3)	34 (18.4)	
Educational background			
University and higher	138 (59.2)	142 (76.8)	<.001
Lower levels of education	95 (40.8)	43 (23.2)	
Smoking			
Yes	7 (3.0)	8 (4.3)	0.471
No	226 (97.0)	177 (95.7)	
Alcohol consumption			
Yes	3 (1.3)	5 (2.7)	.294
No	230 (98.7)	180 (97.3)	
Residence			
Rural	24 (10.3)	7 (3.8)	.012
Urban	209 (89.7)	178 (96.2)	
Household income			
≤10 000 yuan/mo	87 (37.3)	72 (38.9)	.741
>10 000 yuan/mo	146 (62.7)	113 (61.1)	
Causes of RPL			
Parental or fetal factors	138 (59.2)		
Unexplained RPL	95 (40.8)		
Times of spontaneous miscarriage			
2	146 (62.7)	—	—
>2	87 (37.3)	—	—
History of stillbirth			
Yes	33 (14.2)	—	—
No	200 (85.8)	—	—
History of induced abortion			
Yes	154 (66.1)	—	—
No	79 (33.9)	—	—

Values are n (%). A *P* value <.05 was considered as a statistical difference.

Abbreviations: BMI, body mass index; RPL, recurrent pregnancy loss.

### Sexual function of participants

The desire score, arousal score, lubrication score, satisfaction score, and pain score were significantly lower in RPL patients than in healthy women (*P <* .001), but there was no statistical difference in orgasmic score between the 2 groups (*P =* .567). The RPL patients had lower total FSFI scores than healthy women (31.7 [interquartile range, 26.6-33.5] vs 33.0 [interquartile range, 31.2-34.1]; *P <* .001). Compared with healthy women, RPL patients had an increased risk of sexual dysfunction (24.9% vs 8.6%; *P <* .001). These results are shown in Table 2.

**Table 2:** Assessment results of sexual function of participants.

	RPL patients (n = 233)	Healthy women (n = 185)	*P* value
FSFI scores			
Desire scores^a^	5.0 ± 1.0	5.4 ± 0.8	<.001
Arousal scores^a^	5.0 ± 0.8	5.3 ± 0.7	<.001
Lubrication scores^a^	4.9 ± 0.8	5.4 ± 0.7	<.001
Orgasmic scores^a^	5.2 ± 0.7	5.3 ± 0.6	.567
Satisfaction scores^a^	5.0 ± 0.8	5.3 ± 0.8	<.001
Pain scores^a^	5.1 ± 0.8	5.4 ± 0.6	<.001
Total FSFI scores^b^	31.7 (26.6-33.5)	33.0 (31.2-34.1)	<.001
Increased risk of sexual dysfunction	58 (24.9)	16 (8.6)	<.001

Values are mean ± SD or median (interquartile range). A *P* value <.05 was considered as a statistical difference.

Abbreviations: FSFI, Female Sexual Function Index; RPL, recurrent pregnancy loss.

a Continuous variables with normal distributions were compared by Student’s *t* test.

b Continuous variables with abnormal distributions were compared by the Mann-Whitney test.

### Independent association between RPL and increased risk of sexual dysfunction

Because included RPL patients and healthy women were heterogeneous concerning age, educational background, and residence, we performed multivariable logistic regression analysis to explore the independent association between RPL and the risk of sexual dysfunction. We found that RPL was independently associated with the increased risk of sexual dysfunction after adjusting for age, educational background, and residence. RPL patients have 3 times the risk of sexual dysfunction compared with healthy women (adjusted OR, 3.064; 95% CI, 1.665-5.639; *P <* .001). The results are shown in Table 3.

**Table 3:** Multivariable logistic analysis for the increased risk of sexual dysfunction.

	Adjusted OR	95% CI	*P* value
RPL	3.064	1.665-5.639	<.001
Age ≥36 y	1.712	0.917-3.197	.092
Lower levels of education	1.428	0.831-2.452	.197
Live in urban setting	1.026	0.407-2.586	.956

A *P* value <.05 was considered as a statistical difference.

Abbreviations: CI, confidential interval; OR, odds ratio; RPL, recurrent pregnancy loss.

### Risk factors of sexual dysfunction among RPL patients

The risk factors of sexual dysfunction among RPL patients were investigated by univariable analyses and multivariable logistic regression analyses. After univariable analyses, BMI >24 kg/m^2^ (*P <* .001), working >8 h/d (*P <* .029), and unexplained RPL (*P <* .001) were screened to be included in the multivariable logistic regression analysis. We found that BMI >24 kg/m^2^ (adjusted OR, 4.132; 95% CI, 1.902-8.976; *P <* .001), working >8 h/d (adjusted OR, 2.111; 95% CI, 1.020-4.369; *P =* .044), and unexplained RPL (adjusted OR, 3.785; 95% CI, 1.967-7.280; *P <* .001) were independent risk factors of sexual dysfunction for RPL patients. These results are shown in Table 4.

**Table 4:** The risk factors of sexual dysfunction among RPL patients.

	Univariable analysis			Multivariable logistic regression analysis^a^		
	Crude OR	95% CI	*P* value	Adjusted OR	Adjusted 95% CI	Adjusted *P* value
Age ≥36 y	1.347	0.674-2.692	.399			
BMI >24 kg/m^2^	4.312	2.079-8.943	<.001	4.132	1.902-8.976	<.001
BMI <18.5 kg/m^2^	1.522	0.556-4.167	.414	1.460	0.497-4.284	.491
Lower levels of education	1.370	0.753-2.494	.302			
Live in urban setting	0.784	0.308-1.997	.610			
Working hours >8 h/d	2.096	1.077-4.082	.029	2.111	1.020-4.369	.044
Smoking	0.429	0.093-1.975	.277			
Alcohol consumption	0.659	0.059-7.404	.735			
Household income >10 000 yuan/mo	0.724	0.395-1.326	.296			
Unexplained RPL	4.037	2.155-7.564	<.001	3.785	1.967-7.280	<.001
>2 times of pregnancy loss	1.381	0.754-2.528	.296			
History of stillbirth	0.865	0.377-1.987	.733			
History of induced abortion	0.934	0.497-1.755	.831			

A *P* value <.05 was considered as a statistical difference.

Abbreviations: BMI, body mass index; CI, confidential interval; OR, odds ratio; RPL, recurrent pregnancy loss.

a BMI, working hours per day, and causes of RPL were included in the multivariable logistic regression model as independent variables.

## Discussion

Through this multicenter cross-sectional study we found for the first time that RPL patients were at an increased risk of sexual dysfunction compared with healthy women. The association between RPL and sexual dysfunction has been rarely explored previously, and only 1 study exploring the effects of the times of pregnancy loss on sexual function was found. The sexual function of women who have experienced 1 pregnancy loss and those who experienced more than 1 pregnancy loss were also compared, indicating poorer sexual function scores in the latter.^18^ In our study, we assessed the sexual function of participants by the FSFI tool, which was abundantly used in the investigation of female sexual function, and found poorer sexual function scores in RPL patients than in healthy women, too. These results indicated that the influence of RPL on sexual dysfunction should be heavily focused by both patients and clinicians.

We also explored the risk factors of sexual dysfunction among RPL patients. Overweight (BMI >24 kg/m^2^), working >8 h/d, and unexplained RPL were shown to increase the risk of sexual dysfunction for RPL patients. These results may be explained by previous studies. Although the association between overweight/obesity and sexual dysfunction in men was acknowledged,^19^ the relationship between overweight/obesity and female sexual dysfunction was rarely reported before. In recent years, a case-control study found that being overweight and obese seemed to negatively affect sexuality in women with sexual dysfunction,^20^ which was also identified by a systematic review and meta-analysis.^21^ We found the effect of overweight on female dysfunction may be generalizable to RPL patients. Besides, we conclude that working >8 h/d increased the risk of sexual dysfunction in RPL patients, which may be explained by fatigue during work. The relationship between working hours and fatigue was well studied in people with various occupations according to previous studies. Undoubtedly, longer working hours increased cumulative fatigue.^22^^,^^23^ Chronic fatigue was also proved to be a risk factor for sexual dysfunction according to some reports.^24^ However, we found no reference to the role of unexplained RPL on sexual function, in our opinion, this influence may be mediated by some psychological reasons because patients who could not find exact reasons may be more anxious or stressed. This guess needs to be investigated in future research.

This present study provided some clinical guidance for preventing sexual dysfunction in RPL patients. In clinical practice, the status of sexual function should be paid more attention to in RPL patients who are overweight, who have fatigue caused by work, and who have unexplained RPL. Early screening of sexual dysfunction by simple tools such as the FSFI may be beneficial for RPL patients with the previously mentioned risk factors.

### Strengths and limitations

The strength of our study is that it is the first in the world, to our knowledge, to report on the association between RPL and female sexual dysfunction. The study is a multicenter study and was conducted at 3 different hospitals. So, our results were representative of at least a large region of China. A further strength of this study is that the risk factors of sexual dysfunction among RPL patients were investigated, and clinicians can perform early screening of sexual function for patients with these risk factors. The results of our study benefit various RPL patients in clinical practice.

The major limitation of this study is that the cross-sectional design could not confirm the causal relationship between RPL and sexual dysfunction, and even though the diagnosis of RPL was already made when assessing sexual function, we did not know the baseline level of sexual function among these participants. Then, we used the FSFI to evaluate the sexual function of participants; this tool cannot be used by itself to diagnose sexual dysfunction. We can only provide the relationship between RPL and increased risk of sexual dysfunction, not diagnosed sexual dysfunction. In addition, some acknowledged causes of female sexual dysfunction, such as psychological disorders, were not explored in this study; thus, some potential risk factors of sexual dysfunction in RPL patients were not uncovered. The influence of sexual dysfunction on subsequent outcomes in RPL patients was not focused on, which may restrict the value of this study. Prospective cohort studies with a larger sample size will provide more evidence on this topic in the future.

## Conclusion

RPL was significantly associated with an increased risk of female sexual dysfunction, and BMI >24 kg/m^2^, working >8 h/d, and unexplained RPL were risk factors of sexual dysfunction for RPL patients. Accordingly, RPL patients who are overweight, have fatigue caused by work, and have unexplained RPL should be focused on regarding the risk of sexual dysfunction.
